# Harnessing the Heterogeneity of Prostate Cancer for Target Discovery Using Patient-Derived Explants

**DOI:** 10.3390/cancers14071708

**Published:** 2022-03-28

**Authors:** Margaret M. Centenera, Andrew D. Vincent, Max Moldovan, Hui-Ming Lin, David J. Lynn, Lisa G. Horvath, Lisa M. Butler

**Affiliations:** 1 Adelaide Medical School, University of Adelaide, North Terrace, Adelaide, SA 5000, Australia; andrew.vincent@adelaide.edu.au (A.D.V.); lisa.butler@adelaide.edu.au (L.M.B.); 2 Freemasons Centre for Male Health and Wellbeing, University of Adelaide, North Terrace, Adelaide, SA 5000, Australia; 3 Precision Medicine Theme, South Australian Health and Medical Research Institute, North Terrace, Adelaide, SA 5000, Australia; david.lynn@sahmri.com; 4 Biometry Hub, Faculty of Science, University of Adelaide, Waite Campus, SA 5005, Australia; max.moldovan@adelaide.edu.au; 5 Garvan Institute for Medical Research, Darlinghurst, NSW 2010, Australia; h.lin@garvan.org.au (H.-M.L.); lisa.horvath@lh.org.au (L.G.H.); 6 Flinders Health and Medical Research Institute, Flinders University, Bedford Park, SA 5042, Australia; 7 Chris O’Brien Lifehouse, Camperdown, NSW 2050, Australia; 8 University of Sydney, Camperdown, NSW 2006, Australia

**Keywords:** prostate cancer, patient-derived explant, pre-clinical tumor model, transcriptomics, proteomics

## Abstract

**Simple Summary:**

There is a widespread push toward more biologically relevant pre-clinical models of prostate cancer that can improve the discovery and translation of new drugs and biomarkers for this disease. Patient-derived explant culture is an innovative pre-clinical model that utilizes surgical prostate cancer specimens in a way that retains the architecture, microenvironment and heterogeneity of prostate tumors—factors that critically influence cell behavior and response to therapy. With increasing tissue complexity comes increasing complexity of analysis. The aim of this study was to provide critical information for the successful application and analysis of the patient-derived prostate cancer explant model.

**Abstract:**

Prostate cancer is a complex and heterogeneous disease, but a small number of cell lines have dominated basic prostate cancer research, representing a major obstacle in the field of drug and biomarker discovery. A growing lack of confidence in cell lines has seen a shift toward more sophisticated pre-clinical cancer models that incorporate patient-derived tumors as xenografts or explants, to more accurately reflect clinical disease. Not only do these models retain critical features of the original tumor, and account for the molecular diversity and cellular heterogeneity of prostate cancer, but they provide a unique opportunity to conduct research in matched tumor samples. The challenge that accompanies these complex tissue models is increased complexity of analysis. With over 10 years of experience working with patient-derived explants (PDEs) of prostate cancer, this study provides guidance on the PDE method, its limitations, and considerations for addressing the heterogeneity of prostate cancer PDEs that are based on statistical modeling. Using inhibitors of the molecular chaperone heat shock protein 90 (Hsp90) as an example of a drug that induces robust proliferative response, we demonstrate how multi-omics analysis in prostate cancer PDEs is both feasible and essential for identification of key biological pathways, with significant potential for novel drug target and biomarker discovery.

## 1. Introduction

Tumor-derived cell lines cultured in vitro or xenografted into mice are the most widely used pre-clinical model for cancer research. While cell lines are easy to propagate and manipulate, and will continue to be an important resource for basic tumor biology, the translation rate of new oncology drugs from cell line-based research is devastatingly low at ~5% [1]. This statistic makes it clear that cell lines are limited in their predictive power for drug and biomarker discovery research. For prostate cancer, cell line-based models are particularly limiting as isolated primary cells do not survive in culture long term [2], and the field has relied on cell lines, traditionally DU145, PC3 and LNCaP [3]. These cell lines were derived from metastases, therefore do not represent primary disease, and they harbor genetic alterations that are not frequently observed in the clinic. Furthermore, there is now strong evidence that prostate cancer develops through reciprocal interaction between epithelial cells and their surrounding microenvironment, consisting of a diverse structural and cellular network of blood vessels, extracellular matrix, fibroblasts, immune cells and stem cells [4,5]. Prostate cancer cells cultured in isolation are deprived of this critical signaling with the tumor microenvironment. Cell lines are also unable to capture the extensive intra- and inter-patient heterogeneity of clinical prostate cancer that has been evident at the molecular level, via sequencing efforts that revealed a diverse range of prostate cancer subtypes and extensive genomic and transcriptomic aberrations [6,7,8,9].

Given the above limitations, there has been a widespread push toward patient-derived models that more accurately reflect tumors existing within the patient. Compelling evidence for this shift is the United States National Cancer Institute’s discontinuation of its panel of 60 human cell lines (NCI-60), in favor of a rejuvenated repository of cancer models derived from fresh patient samples [10]. A range of patient-derived models exist for prostate cancer, each with their unique advantages and disadvantages [11]. Three-dimensional (3D) culture models, including spheroids and organoids, involve dissociation of the patient tumor into single cell populations that are grown in the presence of a scaffold that facilitates 3D growth in gland-like structures [12]. These techniques have markedly improved the ability to establish and maintain primary prostate cancer cells, but are limited in that components of the microenvironment are still missing and the success rate for prostate cancer organoids is low at ~20% [13]. Patient-derived xenograft (PDX) models, wherein primary human tumor specimens are directly transplanted into immunodeficient mice, are advantageous in that they retain the original architecture and microenvironment of the tumor [14,15]. Importantly, close correlation between PDX sensitivity and clinical response in patients from which the PDX were derived has been demonstrated [16,17,18,19], but again the take rate for prostate cancer PDX is extremely low, the technique is time consuming, costly and tends to favor a limited spectrum of metastatic disease rather than primary disease. 

Over the past decade, our group and others have utilized a patient-derived explant (PDE) technique that, similar to PDX models, utilizes primary human tumors but instead of transplanting the tissue into mice they are cultured on a gelatin sponge that sits in media, reviewed in [20]. PDE culture is much more high-throughput than PDX but still captures the heterogeneity, architecture and microenvironment of prostate tumors [11]. While these complex tumor features are desired for more accurate drug and biomarker development, they make downstream analysis inherently more challenging. Through our evaluation of many different molecular targeted therapeutics and chemotherapies for prostate cancer [21,22,23,24,25,26,27,28,29], some of which directly initiated Phase II clinical trials [30], we have encountered the limitations and considerations needed when using highly heterogeneous prostate tumors. This study presents the critical aspects of patient-derived explant culture and data analysis and provides an example of how PDEs can be used for novel target discovery using an integrated omics approach. 

## 2. Materials and Methods

### 2.1. Patient Consent and Tissue Collection

The urologist made initial contact with patients regarding donation of tissue for research purposes and provided information and consent forms approved by the appropriate ethical regulatory committee. For this study, ethical approval was obtained from St Andrews Hospital (Adelaide, SA, Australia) and St Vincent’s Private Hospital (Darlinghurst, NSW, Australia). Tumors from four independent cohorts of patients were used in this study. Clinicopathologic characteristics of each cohort are detailed in Supplementary Table S1.

Following surgical removal of the prostate, either by robotic radical prostatectomy or transurethral resection of the prostate (TURP), the prostate of consenting patients was transported to pathology on ice to keep it cold but not frozen. Within 30–60 min of surgery, the pathologist used the patient’s diagnostic biopsy results to locate malignant regions. A 4–8 mm skin punch biopsy through the prostatic capsule was used to provide a fresh tissue sample of approximately 10–20 mm in length. The size of the sample taken was determined by the pathologist, typically according to prostate size, as the overall integrity of the prostate must be maintained for diagnostic purposes. Although our method was designed to collect specimens containing malignant foci, there is no guarantee that the tissue obtained contains tumor cells. In our experience, the above method yields a success rate of obtaining tumor tissue of approximately 70%, a factor that must be taken into consideration when forecasting time and budget for PDE-based projects. Upon provision by the pathologist, samples were placed into a vial containing 5 mL cold phosphate buffered saline (PBS) or culture medium and transported to the laboratory on ice.

### 2.2. Explant Tissue Culture

**Figure 1:**
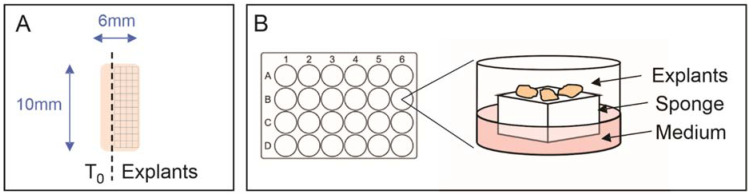
Schematic patient-derived explant culture diagram. (**A**) Fresh prostate tumor specimens obtained from surgery are dissected longitudinally to obtain an uncultured sample at time zero (T_0_). The remaining sample is dissected into 1 mm^3^ pieces for explant culture. (**B**) Explants are cultured on gelatin sponges sitting in medium in 24-well plates, where each well is dedicated to a specific treatment and/or endpoint and contains a minimum of triplicate explants.

All steps were performed under sterile conditions in a biohazard hood. The first step was to soak 10 mm^3^ gelatin sponges (Spongostan–Ethicon Inc., Raritan, NJ, USA; Gelfoam–Pfizer Inc., Brooklyn, NY, USA) in RPMI 1640 medium supplemented with 10% fetal calf serum, 1xantimycotic/antibiotic solution (Sigma, St. Louis, MO, USA), 10 µg/mL hydrocortisone (Sigma), and 10 µg/mL insulin (Sigma). Treatments used include 17-AAG (500 nM), AUY922 (500 nM), enzalutamide (10 µM) and DMSO was used as vehicle control. One sponge and 1 mL of medium was prepared for each well to be used. The sponges were gently agitated in the medium so that all sides were wet, and then soaked for no longer than 5–10 min, otherwise they start to shrink. While the sponges were soaking, the prostate specimen was placed onto a sterile petri dish along with the 5 mL of PBS or media it was transported in, to keep the tissue wet. Using a disposable sterile scalpel, one longitudinal section of the core, approximately 1 mm in diameter, was cut (Figure 1A) and placed into 10% neutral buffered formalin for paraffin embedding. This tissue was called T_0_ and used to determine the presence and percentage of malignant cells in the specimen following staining with hematoxylin and eosin (H&E) and PIN4 triple stain. PIN4 is a clinically used stain, consisting of a cocktail of antibodies directed to Amethylacyl CoA racemase (AMACR), p63 and high molecular weight cytokeratin (HMWK), which are used to distinguish prostate adenocarcinoma (AMACR positive and p63+HMWK negative) from benign prostate glands (AMACR negative and p63+HMWK positive) [31,32]. After obtaining the T_0_ sample, the remaining tissue was dissected into 1 mm^3^ pieces, called explants (Figure 1A). There was usually sufficient tissue to dissect 20–30 explants from a single radical prostatectomy specimen. A critical factor in maintaining tissue morphology and integrity is the size of the dissected tissue as explants that are too small or too large undergo significant necrosis. The tissue culture plate was prepared once the specimen had been dissected, by adding 500 µL treatment media and a single soaked sponge to corresponding wells of a 24-well plate. The treatment media was taken from the container the sponges were being soaked in, and tweezers used to very carefully handle the corners of the sponge and ensure it was not squashed when being transferred to the plate. Tweezers were used to gently transfer explants from the Petri dish onto the sponge, each of which fits a maximum of 4 explants. In a typical experiment, each well of a 24-well plate is dedicated to a specific treatment and/or endpoint and contains a minimum of triplicate explant samples (Figure 1B). The plate was incubated at 37 °C, 5% CO_2_ for the desired period of time, after which, explants were harvested and stored according to desired downstream analysis. For histological analysis, explants were formalin-fixed and paraffin embedded. For RNA analysis, explants were stabilized in RNAlater^®^ (Ambion, Austin, TX, USA) at 4 °C overnight and then stored at −80 °C. For protein or lipid analysis, explants were snap frozen and stored at −80 °C. Conditioned explant media from replicate wells were combined, snap frozen and stored at −80 °C.

### 2.3. Histology

All samples, both T_0_ and cultured explants, were routinely stained with H&E. Formalin-fixed paraffin-embedded sections (2 µm) were heated to 62 °C for 2 h and then de-paraffinized in xylene, dehydrated in ethanol, and hydrated in tap water prior to staining in concentrated Lillie–Meyers hematoxylin for 4 min. Slides were rinsed in tap water, differentiated in 0.3% acid alcohol, blued in running tap water and counterstained with eosin. Slides were again rinsed in tap water before dehydrating in ethanol, clearing in xylene and then fixed with DPX mounting media (Sigma, St. Louis, MO, USA). 

### 2.4. Immunohistochemistry

Immunostaining of formalin-fixed paraffin-embedded sections (2–4 μm) was performed with the Bond RX automated stainer (Leica Biosystems, Wetzlar Germany). Antigen retrieval was 20 min at 100 °C using the Bond Epitope Retrieval solution 2 (EDTA based buffer, pH 9). Incubation with the PIN4 cocktail of antibodies was 1 h at concentrations of 1:200 AMACR (p504S) rabbit antibody (Clone 13H4, DAKO, Glostrup, Denmark), 1:100 p63 mouse antibody (Clone DAK-p63, DAKO, Denmark), and 1:200 34βE12 HMWK mouse antibody (Clone 34BETAE12, Leica Biosystems, Germany). Detection was performed with ChromoPlex 1 Dual Detection (Leica Biosystems), with incubation times of 15 min each for the poly-horseradish anti-mouse IgG conjugate and poly-alkaline phosphatase anti-rabbit IgG conjugate. The first substrate chromogen, 3–3′-diaminobenzidine (DAB), was used to stain basal cells and nuclei of normal glands and prostatic intraepithelial neoplasia (PIN) (brown precipitate for mouse antibodies). Fast Red was applied as the second substrate chromogen to stain the cytoplasm of the adenocarcinoma cells red (red precipitate for rabbit antibodies). Ki67 was used as a marker of proliferation. Slides were incubated 1:200 with Ki67 primary antibody (Clone MIB-1, DAKO, Denmark) for 1 h, followed by goat anti-mouse IgG biotinylated secondary antibody (DAKO, Denmark). Detection was performed using DAB chromogen (Sigma, MO, USA) and counterstained with hematoxylin. In all runs, transurethral resection of the prostate (TURP) tissue was used as the positive control, and explant tissue with no primary antibody used as the negative control. Percent positivity was quantified manually by a specialist prostate cancer pathologist who was blinded to the treatments. Statistical analysis of Ki67 staining was carried out using GraphPad Prism software v7.02 (2016, GraphPad Software, San Diego, CA, USA). Box plots represent the mean ± interquartile, and whiskers represent minimum and maximum values. Statistical significance was determined using one-way analysis of variance (ANOVA). A *p*-value ≤ 0.05 was considered statistically significant.

### 2.5. Statistical Modeling of Ki67 Immunostaining Cell Positivity

While it is appropriate to assume that Ki67 cell positivity in tumors from different patients is independent, the positive cell counts from different areas, or fields-of-view (FoV), within a tumor are not. To determine what distribution best describes these intra-tumor cell counts, we compared Akaike information criterion (AIC) using mixed effects models with different error distributions. In particular, we evaluated the binomial, beta-binomial, Poisson, generalized Poisson, and negative binomial (both with the variance related linearly and quadratically with the mean) distribution. For all models except binomial and beta-binomial, log link was employed and the log transformed total count was included as an offset. In the binomial and beta-binomial models the logit link was employed. In all models, treatment (control vs. active treatment) and batch (samples assessed in four batches) were included as fixed effects. Random intercepts were included for each sample nested within each individual. 

Having determined the model of best fit (above), we used the model to determine the appropriate sample size for evaluating Ki67 staining data. Parameters were set from the observed data, including mean % Ki67 positivity in control and treated samples, and variation across both patients and samples. Data were simulated assuming 25 individuals each with two samples, control vs. treated, and an assumed reduction in Ki67 cell positivity due to active treatment of 0.2 on the logit scale, corresponding to a reduction of approximately 4%. The number of FoVs were varied from 10 through 25 by increments of 5, and the number of cells counted per FoV were 10, 25, 50, 75, and 100. For each combination of FoV count and cell count, 2000 simulations were performed and analyzed using the optimal mixed effects model (as determined above). For each parameter setting, we report the probability of detecting the treatment effect, i.e., the power of the analysis. Statistical modeling and analyses were performed using R packages glmmTMB (v1.0.2.1) and bbmle (v1.0.24).

### 2.6. RNA Sequencing Analysis

Differentially expressed (DE) transcripts in Hsp90 inhibitor treated PDEs were identified by RNA sequencing, as described in our previous report [33]. Proteins in Hsp90 inhibitor treated PDEs were detected using liquid chromatography tandem mass spectrometry (LC-MS/MS) as previously reported [34], and subjected to statistical analysis to detect proteins that were differentially abundant (DA) using a voom-eBayes approach under the paired design (R 3.5.0, limma 3.38.0) [35]. The threshold for statistical significance was set at a false discovery rate (FDR) <5%. FDR was calculated using the Benjamini and Hochberg (1995) method. Principal component analysis (PCA) was performed using the prcomp function from the R stats package. The corresponding PCA plots were drawn using the ggbiplot function from the R ggbiplot 0.55 package. Cross-tissue correlation heat maps were generated using the ggplot2 (version 3.1.0) R package. After matching proteins to their gene IDs, pathway over-representation analysis (ORA) was conducted against the Molecular Signatures Database v6.1 [36]. To assess statistical significance in the pathway analysis, a hypergeometric test was implemented using a one-sided Fisher exact test (R stats package fisher.test function). Molecular Signatures Database “Hallmark” pathways with an FDR <5% were considered to be significantly over-represented. 

## 3. Results

### 3.1. Histology of Patient-Derived Explants Enables Visualization of the Tumor Microenvironment

**Figure 2:**
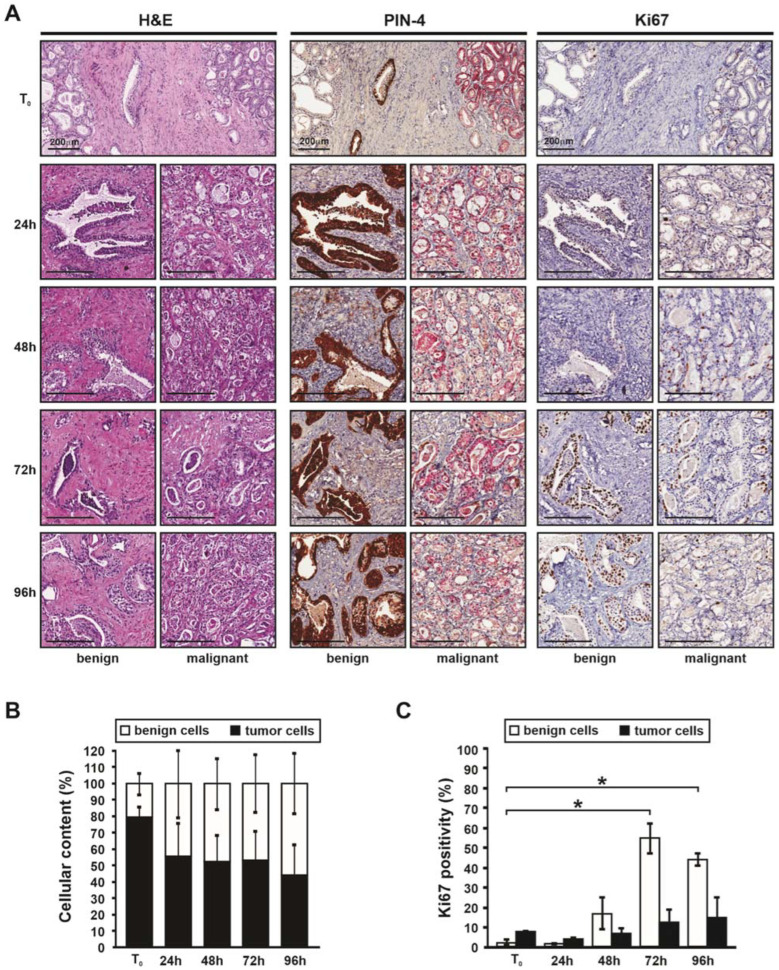
Patient-derived explant (PDE) culture preserves primary tumor characteristics. (**A**) Maintenance of intra-tumoral heterogeneity and tissue viability in PDEs after the indicated culture periods as determined by hematoxylin and eosin staining (H&E), benign and malignant glands identified by PIN-4 prostate triple stain, and tumor cell proliferation by Ki67 staining. Scale bars 200 µm. (**B**) Prostate cancer PDEs (*n* = 4) cultured for 48 h were evaluated for percent glandular tissue containing tumor and benign areas. Data is presented at mean ± SEM of triplicate samples. (**C**) Ki67 positivity in prostate cancer PDEs (*n* = 4) was evaluated in benign and tumor cells. Data is presented as mean ± SEM triplicate samples. ANOVA, time points versus T_0_, * *p* < 0.05.

Fresh primary prostate cancer samples obtained from patients undergoing radical prostatectomy were explanted and cultured for up to 4 days, with tissue harvested for histological analysis every 24 h. Hematoxylin and Eosin (H&E) staining demonstrated that the tumor architecture and cellularity of PDEs was maintained over the entire culture period, as compared with matched uncultured (T0) tumor specimens (Figure 2A). This window of time is another major consideration for PDE culture, to prevent adaptation to ex vivo conditions and loss of cellularity that is observed in tissues cultured longer than 7 days (data not shown). The short time frame of PDE culture makes it high-throughput for measuring intrinsic sensitivity or resistance to therapeutic agents, but limits capacity to evaluate acquired resistance using this model. Within the recommended PDE culture time frame, a heterogeneous mix of cell populations that comprises the tumor microenvironment can be observed, including basal and luminal epithelial cells, immune cells, muscle cells, and stromal cells. Not only are these cells present, but their distribution within the tumor microenvironment can also be seen, highlighting the intra-tumor heterogeneity of prostate cancer. Additionally visible in approximately 20–30% of PDE tissues, are regions of necrosis that are not present in matched T0 tissue (Supplementary Figure S1A) and must therefore represent a response to culture conditions. To minimize necrosis, optimization of media, serum and other additives used in culture is critical at the initial stages of setting up PDE culture for any given tumor or tissue type. In addition to evaluating tumor content upon initial histological analysis (described below), the presence of necrosis should also be noted and used as exclusion criteria for further analysis if more than half of the tissue is necrotic. PIN4 triple staining of T_0_ and PDE tissues delineates benign from malignant regions, both of which are well conserved (Figure 2A). PIN4 staining was used to approximate the presence and percentage tumor area in PDE tissues (Figure 2B) and facilitate analysis of Ki67 staining in benign and malignant tissue regions. As expected for benign prostatic hyperplasia (BPH) tissues, a higher level of Ki67 positivity was observed in benign hyperplastic cells compared with malignant (Figure 2C). Analysis of Cleaved Caspase-3 (CC3) staining showed no significant change in cell death over the 4-day culture period for either benign or malignant tissue areas (Supplementary Figure S1B).

### 3.2. Incorporating Field-of-View Cell Counts Provides Accuracy in Immunostaining Quantification

**Figure 3:**
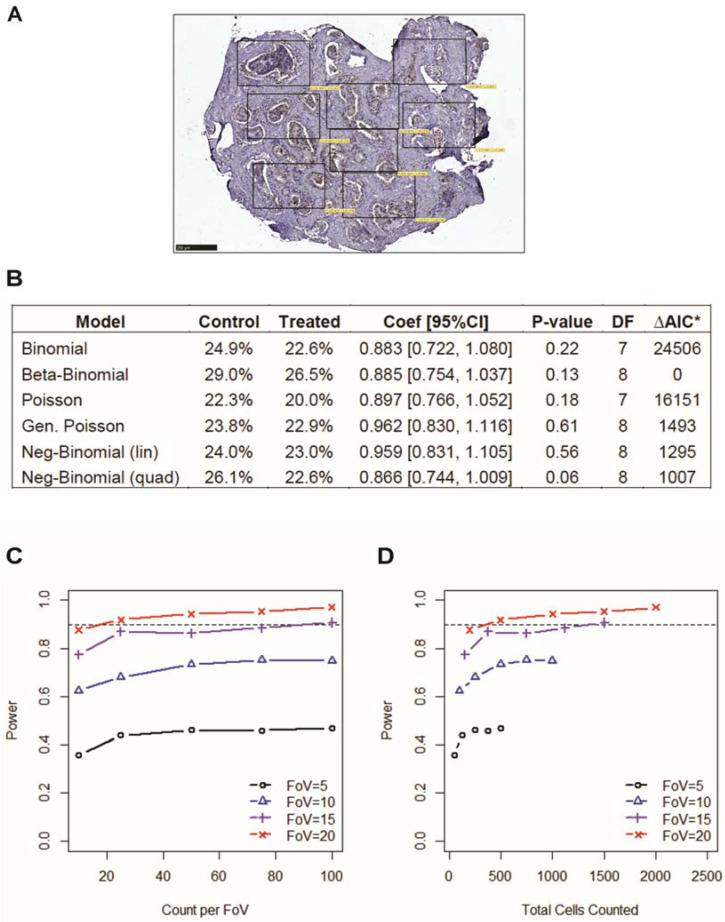
Variation–response modeling of histological cell counts. (**A**) Ki67 cell counts were manually counted across the entire section of PDE tissues using multiple fields-of-view (FoV) for modeling cellular heterogeneity. Each box represents one FoV. (**B**) Mixed effects modeling results for Ki67 immunostaining of PDE tissues cultured in the presence of control or treated with 10 µM enzalutamide (*n* = 122). Coef = coefficient; CI = confidence interval; DF = degrees of freedom; AIC–Aikake information criterion. * ∆AIC is the difference from that of the beta-binomial model, which had the smallest AIC. (**C**,**D**) Ki67 data were simulated assuming a beta-binomial mixed effects model, using set parameters estimated for Ki67. Power is plotted against the number of (**C**) FoV, and (**D**) cell counts per FoV.

As illustrated in Figure 1, PDE tissues are highly heterogeneous in their cellular composition, which means there is underlying variance within and between individual samples that must be considered upon quantitative analysis of immuno-stained explants. To develop a reliable and accurate scoring method for PDE tissues, we undertook statistical modeling of our most widely used immunohistochemistry stain, the proliferative marker Ki67. Ki67 staining is evaluated by counting the number of positively and negatively stained tumor/epithelial cells to calculate the percent of positively stained cells. Cell numbers are manually counted across an entire PDE section using multiple fields-of-view (FoV; Figure 3A), which allows for the modeling of variation across samples. We set out to determine the model of best fit as a way of improving evaluation of staining positivity. Used for analysis were Ki67 positivity data from a cohort of PDEs obtained from 122 prostate cancer patients that were cultured in the presence of vehicle control (DMSO) or clinically used prostate cancer drug enzalutamide (10 µM), as previously described [37]. Mixed effect modeling was applied to the data-set using both total cell count and FoV counts. The different assumptions regarding cell count distributions resulted in variation in the estimate of the mean percent positivity for the average subject. For the control (untreated) samples this ranged from 25% to 32% and from 24% to 29% in enzalutamide treated samples (Figure 3B). The model assuming a beta-binomial distribution provided the best fit with the lowest AIC (Figure 3B). With regards to sample assessment and sample size, the number of FoV had a much larger effect on the power to detect treatment-induced changes in Ki67 positivity, than the number of cells counted per FoV, and must therefore be taken into consideration when analyzing immuno-staining of PDE tissues. For example, there was 80% power to detect a treatment effect of 0.2 (logit scale, 2-sided alpha = 0.05) when at least 25 FoV per sample were assessed with at least 25 cells per FoV, for a total of 625 cells counted (Figure 3C). In contrast, counting 100 cells per FoV but only 10 FoVs, for a total of 1000 cells counted, provided less than 50% power (Figure 3D).

### 3.3. Transcriptomic and Proteomic Analysis of Patient-Derived Explants Identifies Consistent Expression Changes Irrespective of Heterogeneity

**Figure 4:**
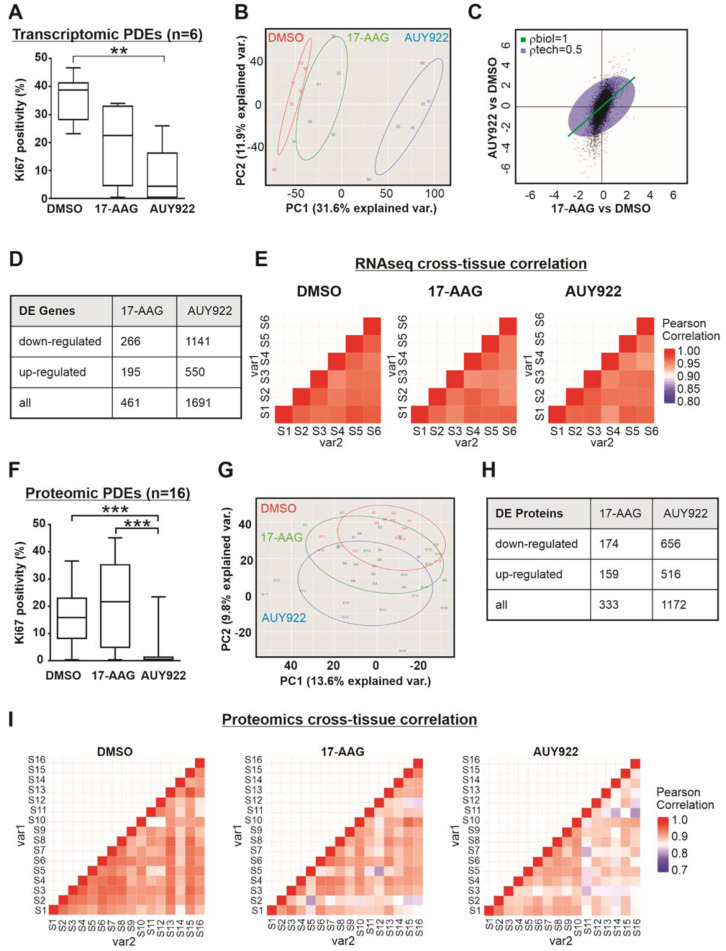
Transcriptomic and proteomic analysis of patient-derived explants. (**A**) Box plot showing significant inhibition of proliferation (Ki67 immunostaining) in AUY922 treated prostate cancer PDEs used for RNAseq analysis (*n* = 6). Box represents the median, 25th and 75th percentile values, and whiskers represent minimum and maximum values. (**B**) Principal component analysis (PCA) of the RNAseq data in A. (**C**) Graphical output from the limma genas function. The plotted log-fold changes indicate that gene expression upon 17-AAG or AUY922 tend to change in the same direction. (**D**) Table summarizing the number of differentially expressed genes identified via RNAseq in prostate cancer PDEs treated with Hsp90 inhibitors. (**E**) Heatmap visualization of cross-tissue correlation in log2-CPM values between all 6 samples assessed for each treatment group. The high positive pairwise correlation coefficients (mean correlations: DMSO = 0.9597, 17-AAG = 0.9546, AUY922 = 0.9551) indicate consistent treatment-related gene expression changes across different biological samples. (**F**) Box plot showing significant inhibition of proliferation (Ki67 immunostaining) in AUY922 treated prostate cancer PDEs used for proteomic analysis (*n* = 16). (**G**) PCA analysis of the proteomic log2-abundance values. (**H**) Table summarizing the number of differentially abundant proteins identified via proteomics in prostate cancer PDEs treated with each inhibitor. (**I**) Heat map visualization of cross-tissue correlation in log2-abundance values for each pair of 16 tissue samples observed for each treatment. The positive pairwise correlation coefficients (mean correlations: DMSO = 0.9083, 17-AAG = 0.8866, AUY922 = 0.8737) indicate consistent treatment-related gene expression changes across the samples. ANOVA, treatment versus DMSO, ** *p* <0.01, *** *p* < 0.001.

The natural variability between PDE samples means that designing omics experiments with adequate power to detect differential expressions at the level required to answer research questions must be carefully considered. A pilot RNAseq experiment was previously conducted and validated [33], and we now reanalyze this dataset to establish the influence of PDE heterogeneity on transcriptomic analysis. PDEs treated with two molecular therapies targeting Hsp90 were used for the pilot RNAseq study, as we have published the proliferative (Ki67) response of prostate PDEs to highly effective Hsp90 inhibitor AUY922 and the less potent inhibitor 17-AAG [22]. PDEs obtained from six prostate cancer patients were cultured in the presence of vehicle control (DMSO), 17-AAG (500 nM) or AUY922 (500 nM) for 48 h, and as previously observed [22], AUY922 significantly suppressed prostate cancer cell proliferation compared to vehicle, whereas 17-AAG did not (Figure 4A). RNA extracted from matched whole PDEs was sequenced using a stranded total RNA approach (Illumina HiSeq, San Diego, CA, USA), which resulted in 32 million reads/sample on average with an alignment rate >90%. Principal component analysis (PCA) of the RNAseq data demonstrated that despite visible inter-patient tissue variation in responses, a significant treatment effect was observed with the most efficacious agent, AUY922 (Figure 4B). Applying the genas (“genuine association”) function in limma [35], the correlation between 17-AAG treatment and AUY922 treatment was evaluated in terms of magnitude and directionality compared to vehicle treatment. Figure 4C plots the log-fold changes for AUY922 vs. vehicle against 17-AAG vs. vehicle, and demonstrates that while gene expression changes with each treatment tended to change in the same direction, the magnitude of change is smaller for 17-AAG than for AUY922, which is consistent with the differential potency of these two agents, illustrated in Figure 4A. Differential expression analysis identified 1691 differentially expressed (DE) genes upon AUY922 treatment (applying a >1.8 fold change, FDR < 0.05 threshold, listed in Supplementary Table S2A), which is 3.7 times more than the 461 DE genes identified upon 17AAG treatment (applying a milder >1.2 fold change to account for the milder potency of the drug, FDR < 0.05 threshold), with the majority of gene expression changes being downregulation compared to vehicle treatment (Figure 4D). Correlation analysis of gene expression between PDE samples demonstrated strong positive pairwise correlations between the biological replicates (Figure 4E). The average correlation coefficients were 0.9597 for DMSO, 0.9546 for 17-AAG and 0.9551 for AUY922, indicating consistent and systematic treatment-related gene expression changes across the six PDE samples. This pilot RNAseq data was utilized as input into Scotty [38], a web-based tool for estimating statistical power in RNAseq experiments. Scotty estimated we would have >90% power to detect a statistically significant change in gene expression of at least 1.5 log-fold between two groups with a similar treatment effect size to AUY922 with a sample size of *n* = 15. An independent cohort of PDE samples obtained from 16 prostate cancer patients was cultured in the absence or presence of either 17-AAG or AUY922 for 48 h, achieving very similar proliferative outcomes to the RNAseq cohort above (Figure 4F). Using proteomics data previously generated and validated from this cohort of PDEs [34], we again re-analyzed the dataset to establish the influence of PDE heterogeneity on proteomic analysis. PCA revealed a significant treatment effect with the more potent AUY922 compared to both control and 17-AAG (Figure 4G). AUY922 treatment resulted in 3.5 times as many differentially abundant proteins as 17-AAG (1172 DA proteins with AUY922, listed in Supplementary Table S2B; 333 DA proteins with 17-AAG) most of which were downregulated (Figure 4H). Cross-tissue correlation in protein abundance was strongly positive, although not as high as our transcript data, with average pairwise correlation coefficients of 0.9083 for DMSO, 0.8866 for 17-AAG and 0.8737 for AUY922 (Figure 4I). Collectively, these results demonstrate that RNAseq and proteomic analysis of PDE tissue is highly feasible, and capable of identifying robust expression changes in response to a drug treatment, irrespective of tissue heterogeneity.

### 3.4. Integration of Transcript and Protein Expression Profiles Identifies Key Pathways of Interest

**Figure 5:**
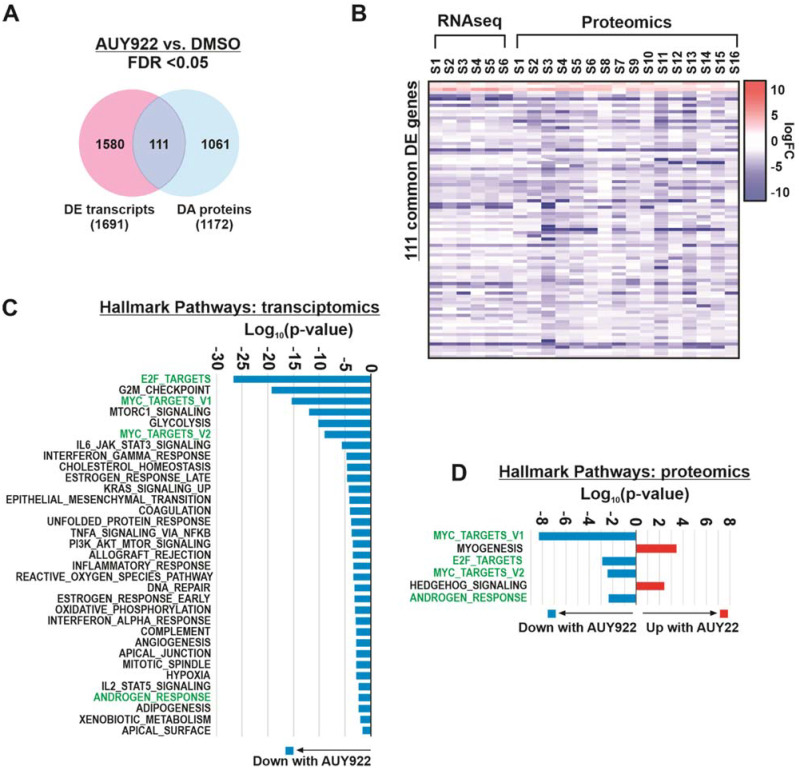
Omics analysis of patient-derived explants identified key biological pathways and novel targets. (**A**) Venn diagram representing the overlap between genes and proteins identified as differentially expressed (DE) or differentially abundant (DA) in AUY922 treated PDEs versus DMSO (FDR < 0.05). (**B**) Heatmap representing 111 genes/proteins that were identified both as differentially expressed in the RNAseq analysis and differentially abundant in the proteomics analysis. (**C**,**D**) Pathway over-representation analysis of (**C**) DE genes and (**D**) DA proteins from Venn diagram in A. Pathways highlighted in green were identified by both RNAseq and proteomics analysis.

Comparison of differentially expressed (DE) transcripts and proteins, in AUY922-treated PDEs compared to vehicle, demonstrated that only a fraction of the individual DE genes/proteins were common between the two datasets (Figure 5A). There were 111 genes/proteins identified as differentially expressed at the transcript level and differentially abundant at a protein level (Supplementary Table S3). Interestingly, all 111 genes/proteins were differentially regulated in the same direction (up or downregulated) in the treated PDE versus the matched control PDE for every transcriptomic (*n* = 6) and proteomic (*n* = 16) sample (Figure 5B). Gene Set Enrichment Analysis (GSEA) of the DE genes identified in the RNAseq dataset identified 33 enriched Hallmark genesets/pathways in AUY922 treated samples (Figure 5C). Six Hallmark genesets were identified as significantly enriched among differentially abundant proteins (Figure 5D). All Hallmark genesets identified in the transcriptomics data were decreased upon AUY922 treatment (Figure 5C). For the proteomics data, four of the six Hallmark genesets were decreased upon AUY922 treatment (Figure 5D) and all four corresponded to Hallmark genesets identified in the transcriptomics data analysis (Figure 5C). This association between RNA and protein profiles provides critical biological validation for omics analysis of heterogeneous PDE tissues. Further, it demonstrates how analysis of multiple datasets facilitates refinement of omics data for identification of key biological pathways. By comparing both transcriptomic and proteomic data, three prominent pathways associated with Hsp90 and prostate cancer were identified, namely MYC targets, E2F targets and androgen response. Two pathways that were stimulated by AUY922 according to proteomics analysis, myogenesis and hedgehog signaling (Figure 5D), were not significantly differentially expressed at the transcript level, which demonstrates that proteomics can reveal novel pathway alterations not detected by transcriptomics.

## 4. Discussion

The search for effective new prostate cancer treatments remains a major challenge due to the significant heterogeneity that is characteristic of prostate cancer but is not reflected in current cell line or animal models. There is an urgent need for more biologically relevant pre-clinical models of prostate cancer that can improve the discovery and translation of new drugs and biomarkers for this disease. PDEs are an innovative tool that capture the complexity of prostate cancer by preserving the native architecture and microenvironment of the tissue. As the interest and uptake of patient-derived models continues to rise, successful application of this culture technique will provide a platform for the development of new drugs, for prediction of clinical efficacy of treatments, and for identification of predictive biomarkers.

Central to any PDE study is a multi-disciplinary collaboration with urologists and pathologists for the collection of high-quality biological samples using consistent and precise protocols. When setting up PDE culture, three key aspects need to be kept in mind for success, these include (1) time, (2) tissue viability, and (3) endpoint analysis. The hands-on time needed to set up a PDE experiment is not extensive compared to working with cell lines, but the amount of time needed to process and evaluate samples for histological and endpoint analysis is considerably more time consuming and varies significantly depending on the number of samples in the study. Moreover, not all tissues can be included for analysis due to lack of viability or insufficient numbers of epithelial/tumor cells, and this typically necessitates 20–30% more tissues to be acquired and evaluated histologically before reaching the desired sample size, which can be one of the most time-consuming and unpredictable aspects of PDE culture. PDE tissue viability is influenced by numerous factors detailed throughout this study, and includes dissection of tissues into the correct size, use of aseptic technique, optimized culture medium, and short-term timeframes to minimize adaptation. An increase in necrosis can be seen in PDE tissues when these aspects are not upheld. Necrotic tissues are not suitable for endpoint analysis and it is important that PDEs containing significant necrosis are excluded from further investigation. Post-cultivation histological analysis must therefore be conducted to select PDEs with good viability and tumor content for robust endpoint analysis. The heterogeneity of prostate tumor PDEs is their major advantage, but also their major challenge in terms of endpoint analysis. Inclusion of biological replicates is an absolute requirement for addressing heterogeneity as it ensures that a sufficient number of epithelial/tumor cells is present for analysis, but also allows for the use of FoV in evaluating immunostaining of PDEs. Our modeling demonstrated that the number of FoVs counted had more influence on power than the total number of cells counted, therefore incorporating FoV counts improves accuracy of evaluating drug efficacy. Given that efficacy studies are often the first PDE experiments with any given drug, and evidence of significant drug efficacy is typically a gateway to further evaluation into mechanism, resistance, biomarker development and selection for clinical trials, having high confidence in these initial tumor responses is critical. Robust treatment efficacy data is also key to successful PDE-based omics experiments and should form the basis of experimental design.

Hsp90 is a molecular chaperone that is responsible for the folding and function of over 300 “client” proteins involved in prostate cancer development and progression; thus, targeting Hsp90 results in broad inhibition of critical signaling pathways (reviewed in [39,40]). Hsp90 has been extensively investigated as a target in prostate cancer both pre-clinically and clinically [41], but Hsp90 inhibitors such as 17-AAG and AUY922 have not yet been clinically approved despite continuing efforts [42]. 17-AAG was the first-in-class Hsp90 inhibitor, and it has far less potency than 2nd generation agents such as AUY922 [22]. We detected the differential potency between these drugs, not only through differences in the proliferative response of PDEs, but through the magnitude of gene/protein expression changes in response to each treatment. We also identified three pathways, androgen response, MYC targets and E2F targets, that were commonly inhibited at both the transcript and protein levels. These are well established oncogenic pathways in prostate cancer, driven by transcription factors AR, MYC and E2F1/2. AR is undeniably one of the most critical drivers of all stages of prostate cancer development, progression and treatment resistance, and it has been the mainstay of prostate cancer treatment for decades [43]. Another major driver of prostate tumorigenesis and progression, MYC expression correlates with increased disease severity and it is frequently mutated in prostate cancer, making MYC an intensely researched therapeutic target [44]. E2F1 has been attributed to numerous cancer hallmarks in prostate cancer, including cell cycle, proliferation and apoptosis [45], and its expression is so closely aligned with disease stage that it has been proposed as a potential biomarker [46,47]. Notably, AR, MYC and E2F1/2 are all established Hsp90 client proteins, and targeting Hsp90 inhibits expression of AR in prostate cancer (reviewed in [41]), and MYC [48,49,50,51] and E2F1 [52,53] in other cancers. Our ability to identify known pathways associated with Hsp90 and prostate cancer through omics analysis of PDE tissues highlights the capacity for omics identification of novel targets and biomarkers in the face of prostate tumor heterogeneity.

What remains to be seen is whether prostate cancer PDE data will correspond with patient data. Studies in other cancers suggest that there will be a strong correlation. In both ovarian cancer [54] and lung cancer [55], PDE sensitivity to chemotherapy was positively associated with survival in clinical trial patients. We are actively seeking to validate our published prostate cancer PDE data that showed efficacy of CDK4/6 inhibitors [23], through a pharmacodynamic neoadjuvant clinical study of CDK4/6 inhibitors in prostate cancer [30]. Demonstrating that our clinical data mirrors pre-clinical PDE results will provide irrefutable evidence for the use of PDEs in drug and biomarker discovery. It will also support recent studies suggesting PDEs as a platform to directly inform clinical management through precision medicine [56].

## 5. Conclusions

Patient-derived models hold significant promise for translational research and represent a new era in prostate cancer research. Similar to all models, PDEs have limitations that mostly include lack of an immune system, although immune cells are indeed present with the tumor environment of PDEs, and inability to evaluate acquired resistance due to adaptation to culture conditions within 7 days. Despite these limitations, the fact that they are actual tumors from patients that are cultured and treated in the laboratory makes PDEs the most biologically relevant pre-clinical representation of the heterogeneity and diversity of prostate cancer. Harnessing the complexity of PDEs to guide the development and implementation of new drugs and biomarkers will lead to better management of prostate cancer and ultimately improve patient survival and quality of life.

## Data Availability

The data presented in this study are available on request from the corresponding author.

## Supplementary Materials

The following supporting information can be downloaded at: https://www.mdpi.com/article/10.3390/cancers14071708/s1, Table S1: Clinicopathologic characteristics of patients included in this study; Table S2A: Differentially expressed genes in AUY922 treated PDEs versus vehicle treated PDEs; Table S2B: Differentially expressed proteins in AUY922 treated PDEs versus vehicle treated PDEs; Table S3: List of 111 common genes/proteins; Figure S1A: Histology of H&E demonstrating necrosis in PDEs; Figure S1B: Cleaved Caspase-3 staining of PDEs.
