# Supplementary material for: Harnessing the Heterogeneity of Prostate Cancer for Target Discovery Using Patient-Derived Explants
Source: Cancers (Basel). 2022 Mar 28;14(7):1708. doi: 10.3390/cancers14071708 (PMC8996971; doi:10.3390/cancers14071708)
Supplement: Supplementary file 1 [file cancers-14-01708-s001.zip › Supplementary Figure S1.pdf]

Supplementary Figure S1

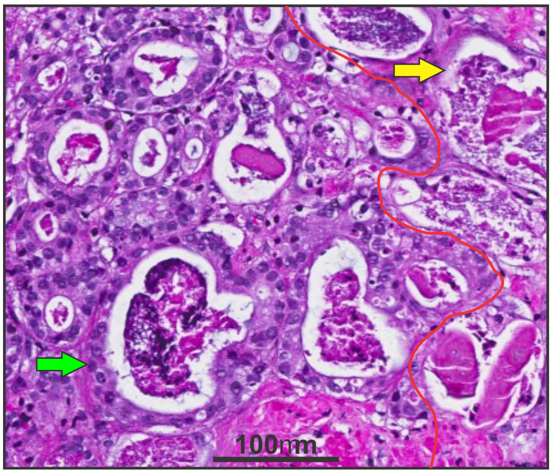

Supplementary Figure S1A. Histology of H&E stained prostate cancer PDE cultured for 72h. Red line demarcates viable region on the left and necrotic region on the right. Green arrow in the viable region points to epithelial cells with nuclei present that line the glandular structure. Yellow arrow in the necrotic region points to architectural structure with no cells/nuclei.

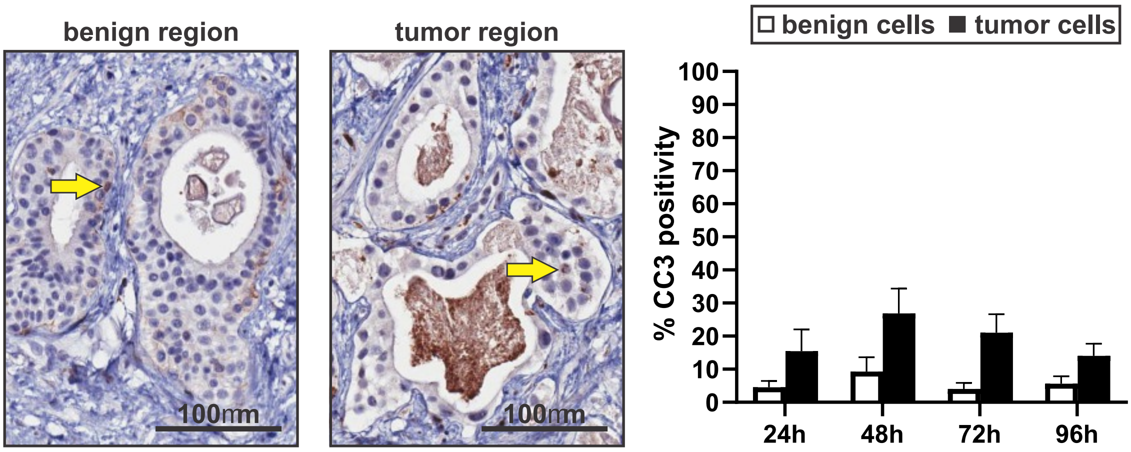

Supplementary Figure S1B. Cleaved Caspase-3 staining examples and analysis of % positivity in benign and tumor regions of prostate cancer PDEs (n=3) cultured for 96h. Yellow arrows point to positively stained cells. Data is presented as mean +/- SEM of triplicate samples. ANOVA; no significance between time points for either benign or tumor cells.
